# Peculiarities of the X-Rays

**Published:** 1897-09

**Authors:** 


### PECULIARITIES OF THE X-RAYS.

Dr. Andrew Wilson in his Science Jottings in the February, 1897, number of the *Illustrated London News*, gives a pleasing suggestion of the possibilities for further usefulness which may perhaps be yet found in the recently discovered power of the Roentgen Rays. The doctor says: "That there is more in the X-Rays than at first sight appears is evinced by the accounts we are receiving of certain peculiar effects these rays exert on the human skin. In one case a patient lost his hair after exposure to the influence of the Roentgen rays, and now we hear of cases in which skin inflammation has been produced by them. The symptoms described by a medical man who had subjected himself for scientific purposes to the new photography were of fairly severe character. These incidents appear to prove that the rays are of singularly powerful nature, and that some useful application or other of the at present undesirable effects may not be at all an unlikely discovery. It would indeed prove interesting if, in addition to their diagnostic powers, a curative action of the rays were noted."—*Montreal Homœopathic Record.*

